# Assessment of Autophagy in Neurons and Brain Tissue

**DOI:** 10.3390/cells6030025

**Published:** 2017-08-23

**Authors:** Irene Benito-Cuesta, Héctor Diez, Lara Ordoñez, Francisco Wandosell

**Affiliations:** 1 Centro de Biología Molecular “Severo Ochoa” (CSIC-UAM), C/Nicolas Cabrera 1, Universidad Autonoma Madrid, Madrid 28049, Spain; irene.benito@csic.es (I.B.-C.); hectordiez81@gmail.com (H.D.); lordoniez@cbm.csic.es (L.O.); 2 Centro de Investigación Biomédica en Red de Enfermedades Neurodegenerativas (CIBERNED), Valderrebollo 5, Madrid 28049, Spain

**Keywords:** macroautophagy, signalling, mTORC1, PI3K, Alzheimer’s disease, proteinopathies

## Abstract

Autophagy is a complex process that controls the transport of cytoplasmic components into lysosomes for degradation. This highly conserved proteolytic system involves dynamic and complex processes, using similar molecular elements and machinery from yeast to humans. Moreover, autophagic dysfunction may contribute to a broad spectrum of mammalian diseases. Indeed, in adult tissues, where the capacity for regeneration or cell division is low or absent (e.g., in the mammalian brain), the accumulation of proteins/peptides that would otherwise be recycled or destroyed may have pathological implications. Indeed, such changes are hallmarks of pathologies, like Alzheimer’s, Prion or Parkinson’s disease, known as proteinopathies. However, it is still unclear whether such dysfunction is a cause or an effect in these conditions. One advantage when analysing autophagy in the mammalian brain is that almost all the markers described in different cell lineages and systems appear to be present in the brain, and even in neurons. By contrast, the mixture of cell types present in the brain and the differentiation stage of such neurons, when compared with neurons in culture, make translating basic research to the clinic less straightforward. Thus, the purpose of this review is to describe and discuss the methods available to monitor autophagy in neurons and in the mammalian brain, a process that is not yet fully understood, focusing primarily on mammalian macroautophagy. We will describe some general features of neuronal autophagy that point to our focus on neuropathologies in which macroautophagy may be altered. Indeed, we centre this review around the hypothesis that enhanced autophagy may be able to provide therapeutic benefits in some brain pathologies, like Alzheimer’s disease, considering this pathology as one of the most prevalent proteinopathies.

## 1. Introduction

The term “autophagy“ was first employed in cell biology around 20 years ago and led to an explosion of information about a group of evolutionarily conserved genes (from yeast to humans) involved in this process. Subsequently, autophagy was implicated in a wide range of homeostatic, developmental and physiological events, and its deregulation has been linked to a wide spectrum of mammalian diseases [1,2,3,4,5]. For instance, in relation to pathologies like Alzheimer’s (AD) or Parkinson’s (PD) diseases, there is substantial evidence from patients and animal models that autophagy is dysregulated [3,6]. A group of neuropathologies can be defined as proteinopathies, whereby defects in autophagy contribute to the accumulation of peptide/protein aggregates. However, it remains unclear whether such defects are the cause or effect of these pathologies.

In most mammalian cells, autophagy is classified into three general subtypes based on how substrates are delivered to the lysosomal compartment: chaperone-mediated autophagy, microautophagy, and macroautophagy [7]. Our laboratory has focused on the role of macroautophagy in neurons and in models of neurodegeneration. This process is essentially a constitutive mechanism responsible for the turnover of cytoplasmic constituents of the cell, and it may be enhanced under conditions of cell injury or nutritional deprivation in order to generate new substrates, thereby complying with the cells energy demands and/or needs for cellular remodelling. Under such conditions, a new double-membraned vacuole is created at phagophore assembly sites (PAS) and is elongated under the direction of two ubiquitination-like reactions: the conjugation of Atg12 to Atg5 by Atg7 proteins; and the conjugation of the cytosolic microtubule-associated light chain 30 (LC3-I) with phosphatidylethanolamine to form the membrane-associated LC3-II [8,9,10]. Together, these proteins orchestrate the sequestering of cytoplasmic proteins into double-membrane-limited autophagic vacuoles (AVs) or autophagosomes through autophagic adaptors, such as the cargo receptors p62/SQSTM1 (sequestosome 1) and NBR1 (neighbour of Brca1 gene: [11,12]. Finally, autophagosomes mature into single-membrane autophagolysosomes by fusing their outer membrane with lysosomes, in which their contents are degraded by acidic lysosomal hydrolases and the lysosome is then restored.

Autophagy involves dynamic and complex processes that are as yet not fully understood. Thus, the purpose of this review is to describe and discuss the methods available to monitor autophagy in neurons and in the mammalian brain, primarily focusing on mammalian macroautophagy (from now on referred to simply as “autophagy”).

## 2. Methods for Monitoring Neuronal and Brain Autophagy

General methods to assay autophagy can be employed in neuronal systems, such as those compiled elsewhere [13]. Numerous autophagy-related markers appear to be altered in different neurodegenerative paradigms, including the conversion of LC3 to LC3-II, NBR1 and SQSTM1/p62 degradation, AMPK/mTORC1 activity, etc. Constitutive autophagy is particularly relevant in neurons and in the brain as a part of normal physiology. Indeed, deficient autophagy caused by the mutation of some proteins may cause neuronal cell death and/or neurodegeneration [14,15,16,17]. However, when studying the role of autophagy in neurons, some specific issues cannot be ignored. On the one hand, it is important to consider whether neuronal cell lines or primary cultures of neurons are being analysed or if the brain itself is under study. On the other hand, it must be noted whether the inhibitors/activators of autophagy used cross the blood–brain barrier (BBB) or not, influencing their effect on the brain tissue. In addition, any potential toxicity of such compounds must also be taken into account. Thus, drawing direct comparisons between neurons and the brain, in physiological or pathological conditions, is not straightforward.

In addition to neurons, the central nervous system (CNS) is composed of astrocytes, microglia, oligodendrocytes and endothelial cells, which in the case of the mouse brain exist in a ratio of about 3:1 glia/neuron [18]. Thus, the relative contribution of these cells has to be taken into account when analysing brain tissue, for example in Western blots, and in many cases comparing data from neurons or glia and brain tissue may be of interest. To gain more detailed information, primary neuron cultures and complementary neuronal lines can be employed, cell systems that allow us to compare autophagic responses under basal conditions or after manipulation with agents that modify autophagosome formation or subsequent degradation steps.

## 3. Autophagy in Neuronal Systems: Neuroblastoma Cell Lines and/or Primary Neuronal Cultures

### 3.1. Basal Autophagy

Neurons are highly polarised cells with two morphological and biochemically distinct compartments that contain specific proteins, a somato-dendritic and axonal compartment [19,20]. This polarity is essential for neuronal physiology, the structural integrity of which relies on efficient proteostasis, and it seems that autophagy is particularly important in maintaining the axonal and dendritic components [21,22]. Autophagy is highly regulated in neurons, which contain few autophagosomes, and may occur within the somato-dendritic compartment, in distal axons or at synaptic terminals [23]. While the AVs that form in the somato-dendritic compartment cannot access the axon, those formed in the distal axon can undergo retrograde transport to the soma [23,24,25]. Indeed, there is evidence that the axon plays an important role in constitutive autophagosome generation and transport [24,26], and that axonal transport is essential to clear damaged mitochondria [26] even though their autophagic degradation may occur locally [27]. Indeed, amphisomes are exceptionally common in the axons of neurons, as distal autophagosomes fuse with endosomes in the pathway towards perinuclear lysosomes [15].

**Figure 1:**
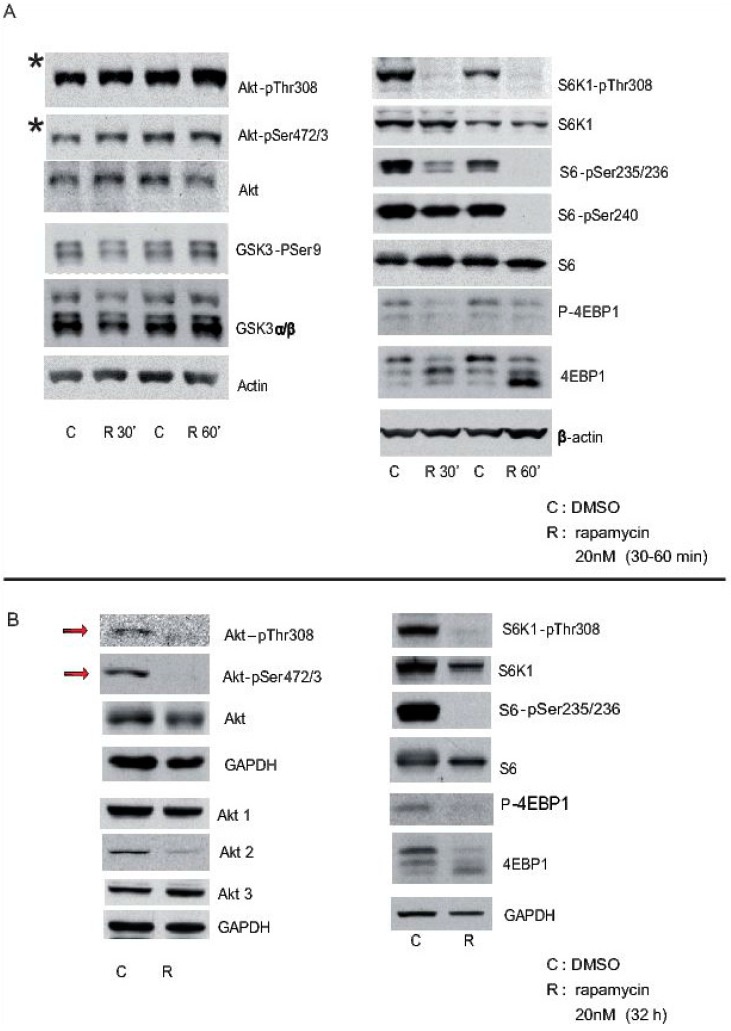
Effects of pharmacological inhibition of mTORC1 on cultured primary cortical neurons. The status of the Akt-mTORC1 pathway was analysed after 30 min or 60 min of rapamycin incubation (**A**); orafter longer period of time (32 h) (**B**). **C** represent the control solvent and **R** represent rapamycin treatment, at each time point analysed.

For many years neuron cell lines have been used as models to analyse autophagy (e.g., SH-SY5Y, Neuro2A, SK-N-SH, etc.), although each have little or no capacity to polarise. Moreover, primary neurons are usually cultured for just 3–5 days in vitro, which may not give them time to become fully polarised. They needs around 2–3 weeks following the Banker´s lab procedure to be fully polarised (for review see. {Bentley, 2016 #235}). It is important to note these differences between neuronal cell lines and primary neurons, as each system may be distinct when assessing specific parameters of autophagy. Indeed, some cell lines are aneuploid [28] or polyploid, which is likely to also cause proteomic discompensation. Thus, comparative analyses of cell lines may identify different responses, as reported for the inhibition of mTORC1 by rapamycin [29,30]. For instance, the degree of autophagy in SH-SY5Y cells, as reflected by the conversion of LC3 to LC3II, appears to be modest when triggered by mTORC1 inhibition, whereas it is more robust when we use the Calpain inhibitor, Calpeptin (unpublished data). One explanation for this effect of rapamycin is that rapamycin mediated inhibition of mTORC1 may also produce time-dependent mTORC2 inhibition in several tumour cell lines, the latter representing an important regulator of Akt phosphorylation [31]. This effect is also evident in primary neurons after 32 h in the presence of rapamycin (Figure 1), although it has not been detected in vivo in brain tissue.

Similarly, when we analysed the basal status of PI3K-Akt-mTROC1 in these neuronal cell lines, the activity of PDK1, Akt or mTORC differed when inferred from the phosphorylation of different substrates. With respect to primary neurons, the degree of polarisation and the source of the neurons may be important, although generally not much attention is paid to these issues. Indeed, some of the elements in the PI3K-Akt pathway are expressed distinctly during maturation, not only during brain development in vivo but also, in cell culture. This is this case of Akt activity, when inferred by threonine 308 and serine 472/3 phosphorylation, and of the different Akt isoforms (as is more evident in Akt2: [32], and as also occurs with LKB1-AMPK in different neuronal lineages. In conclusion, when comparing data regarding autophagy in the brain and isolated neurons, it must be borne in mind that this generally involves a comparison between mature and immature neurons, and that the influence of the glia components present in vivo is absent in culture.

### 3.2. Activators and Inhibitors of Autophagy

The second way to obtain information about autophagy in neurons and neuronal cell lines is through the use of activators or inhibitors that disturb the basal status. In terms of activators, different molecules have been used in diverse cell systems to activate basal autophagy, such as: mTORC1 inhibitors (e.g., rapamycin); some PI3K-mTOR inhibitors like PI-103 or Torin 1; the Calpain inhibitor, Calpeptin; calcium channel blockers such as Nimodipine or Verapamil; or less specific activators like resveratrol, proteasome inhibitors (e.g., MG132) and ER stress drugs (e.g., Tunicamycin or Brefeldin A). Most of these compounds have some effect on neuronal cell lines or primary neurons, although to date there is little comparative data available.

In terms of inhibitors, some drugs that disrupt the cytoskeleton have been reported to inhibit autophagy, such as Nocodazole or Taxol, and paradoxically some PI3K inhibitors like 3-Methyladenine (3-MA), LY 294002 or Wortmannin. This contradiction arises from the fact that activators of autophagy like PI103 or Torin 1 (both PI3K-mTOR inhibitors), may generate similar a biochemical profile to that of LY 294002 or Wortmannin, such as inhibiting Akt or mTORC1 activity, etc. However, these compounds were reported to have the opposite effect in other cell systems: for instance, LY 294002 generates a neuronal polarity defect in our hands that cannot be reproduced by the new PI3K class I inhibitor GDC0941, or by a combination of this PI3K inhibitor and rapamycin [33]. We also detected that the high concentration of 3-MA used when it was described as an inhibitor of PI3K class III and autophagy may also provoke the inhibition of PI3K class I [33].

The way in which autophagic flux is estimated should also be borne in mind. Several compounds have been used to this end, like the vacuolar H^+^-ATPase inhibitor Bafilomycin A1, Chloroquine diphosphate or NH_4_Cl, which will directly affect lysosomal pH, prevent acidification and activate proteases. Alternatively, several general protease inhibitors have been used to block lysosomal degradation, such Pepstatin A or Leupeptin [13,34].

### 3.3. Specific Methods to Monitor Autophagy in Neuronal Systems

Several methods can be used to monitor autophagy in neuronal and non-neuronal cells, although some particularities of each cell type must be taken into account. Firstly, autophagic responses to different stimuli may vary not only between cell types or between dividing and non-dividing cells but also within a specific cell type depending on its state of confluency/differentiation and/or the culture medium or conditions. The most common methods to monitor autophagy in neuronal cell systems are reviewed below:

Transmission electron microscopy (TEM) can be used to characterise the presence of specific autophagic structures. However, when primary neurons or differentiated neuroblastoma cell lines are harvested in culture, their projections or neurites are disrupted, which alters the cell’s morphology. Moreover, some confusion may be generated if differential studies are carried out on neuronal compartments, unless the presence of the nucleus is used to ensure correct identification of the soma. To avoid such issues, samples can be prepared on the surface of coverslips [35]. In aged post-mitotic cells like neurons, the presence of residual bodies has been reported, such as a lysosome subtype that reflects unsuccessful incomplete autolysosomal digestion that can be readily distinguished by TEM [13,36].

LC3-I to LC3-II conversion can be readily assessed in Western blots due to the distinct electrophoretic mobility of these isoforms, or through the LC3 dots that can be detected by immunofluorescence. A high ratio of LC3-I to LC3-II is common in neuronal cells and only a slight increase in LC3-II occurs on inducing autophagy by nutrient deprivation or with chemicals. This feature has been related to an efficient basal autophagic flux, where the enhanced formation of autophagosomes is associated with their rapid clearance by fusion with lysosomes [23]. However, blockage of lysosomal fusion and degradation after different pro-autophagic stimuli only provokes a mild increase in autophagy in our hands, contrasting with the notable increase observed in tumour cell lines. In neuronal cultures, it is especially important to adjust the time and concentration of exposure to some lysosomal inhibitors (e.g., bafilomycin A1) in order to avoid cytotoxicity, and the results should be contrasted to those achieved through other strategies. Given the tight regulation and time restrictions associated with primary neuronal cultures, results based on LC3 conversion may be confusing. As a complementary strategy, assays of lysosomal activity can be performed to define their degradation capacity. In our hands, the lysosomal activity of neuronal cultures can be efficiently measured by assessing cathepsin activity or by in vivo monitoring of the pH-dependent intensity of fluorescent acidotropic dyes.

To avoid the use of lysosome inhibitors, autophagic flux can also be determined via the expression of GFP-LC3B [37] or a tandem mCherry/mRFP-GFP tagged LC3 construct [38]. The assay of the former is based on the LC3 part of the chimera being more sensitive to degradation than GFP, such that both elements can be monitored in Western blots to estimate lysis by lysosomes. The latter assay is based on the GFP fluorescent signal being more sensitive to the acidic conditions of the lysosome, whereas mCherry or mRFP is more stable. Accordingly, the co-localisation of both GFP and mCherry/mRFP fluorescence corresponds to a phagophore or an autophagosome, whereas an mCherry/mRFP signal independent of GFP indicates lysosomal fusion. However, the problems associated with neuronal transfection make it necessary to optimise the protocols when using these techniques. In our hands, neuronal cells show very variable expression of the tandem mRFP-GFP-LC3 construct in culture and very weak mRFP signals, with or without GFP, consistent with the smaller number of autophagic vesicles in neurons, which makes the interpretation of these assays more difficult. More recently, an alternative construct, pHluorin-mKate2-LC3, has been reported that is suitable for monitoring autophagic structures {Tanida, 2014 #320} {Tanida, 2017 #319}.

Although several methods have been described to determine autophagic flux by flow cytometry, this technique is not appropriate for neuronal cells with long projections that would be disrupted when detached from the surface. This technique could be used for non-differentiated neuroblastoma cell lines, such as SH-SY5Y, although the preparation of adherent cells for such assays may induce secondary autophagy [13].

As neurons are highly dependent on healthy mitochondria, mitophagy has been extensively studied in these cells. However, it must be taken into account that certain types of mitophagy induced by BNIP3L/NIX are dependent on gamma-aminobutyric acid receptor-associated protein (GABARAP) and they are less dependent on LC3 proteins [39,40]. Indeed, an enrichment of GABARAP has been described in certain neuronal areas [41] and thus, studying this mammalian homologue of the Atg8 subfamily should be considered.

The rate of autophagic degradation promoted by autophagy can be assessed by studying some adaptor proteins specifically bound to autophagosomes in Western blots. In neurons, measuring the autophagic adaptor NBR1 in flux experiments with lysosomal inhibitors reflects LC3-II turnover, making this a useful complementary tool to assay the rate of degradation. This analysis can be also done with the better studied autophagic adaptor p62/SQSTM1, although the high levels of this protein and its long half-life make it harder to appreciate significant differences with the short exposures to lysosomal inhibitors. However, these features make p62/SQSTM1 an optimal marker to assay any increase in the rate of degradation in our neuronal system after long-term treatment with an inducer of autophagy.

## 4. Autophagy in Mouse Models

The methods available to monitor autophagic flux in vivo have been poorly developed and the obstacles associated with drug bioavailability in the CNS make this an even more difficult task.

### 4.1. Methods to Monitor Autophagy In Vivo

*Electron microscopy (EM).* Dystrophic neurites in some neurodegenerative diseases are rich in autophagic structures that are enriched in hydrolases and that contain partially digested amorphous substrates. These structures are derived from the autolysosomes and damaged lysosomes (“lysosophagy”) characteristic of active autophagy [3]. EM enables the transcellular degradation of mitochondria (“transmitophagy”) to be visualised in the nervous system, as described when astrocytes degrade an axon-derived protrusion containing damaged mitochondria [42]. Like any ex vivo tissue, the brain should be fixed immediately to avoid changes in autophagy, and thus perfusion fixation is recommended.

*LC3-I to LC3-II conversion*. According to our data, LC3-I is much more abundant than LC3-II in brain tissue [43]. To analyse this conversion, the membrane fraction of a cell lysate may need to be purified for immunoblot analysis [44]. To determine the autophagic flux, degradation should be blocked, although most of the usual drugs employed in cell culture have some toxic effects and they must be able to cross the BBB when used in vivo. Leupeptin crosses the blood—retinal barrier and blocks degradation in the retina after intraperitoneal (ip) injection, constituting an interesting model to study autophagy in the CNS [45]. Other strategies involve the intracerebroventricular (icv) injection of leupeptin [46] or the ip injection of the lysosomotropic dye monodansylcadaverine (MDC) that co-localises with LC3 dots [47,48].

*In vivo transgenesis*. Transgenic mice represent an alternative method to analyse autophagy and indeed, GFP-LC3 transgenic mice have been employed to assess the autophagic flux in Western blots of postmortem tissue, the cleavage of GFP-LC3 releasing free GFP [13,43]. However, the accumulation of free GFP in the mouse brain is minimal after inducing autophagy but significant when this process is blocked after traumatic brain injury [49]. Multiple mutant Atg mice have been developed (KOs) in which these proteins are knocked down in the nervous system [17,50,51,52,53,54,55,56,57]. In addition, to limit the transfection of autophagy-related constructs into the CNS, adeno-associated virus can be injected icv into new-born mice. This approach has been used to achieve the in vivo expression of mCherry-GFP-LC3 [58,59] or of Mito-EGFP-mCherry to specifically study mitophagy [42].

### 4.2. Autophagy in Mouse Models of Neurodegeneration

Alterations to autophagy in the nervous system have been associated with some neurodevelopmental, neurometabolic and neurodegenerative disorders [60,61,62,63]. Moreover, in many cases, these defects in autophagy are considered drivers of disease pathogenesis rather than secondary consequences. Indeed, excess autophagy may be detrimental when associated with specific acute neural damage [62,63,64], whereas deficient autophagy might lead to the accumulation of misfolded proteins, which hinders normal cell physiology. Such conditions are considered proteinopathies, as is the case of most late-onset neurodegenerative diseases like AD, PD and Huntington’s disease (HD: the role of autophagy in many neurological diseases has been reviewed recently in [65]). Autophagic and lysosomal activity diminishes with age, and lower rates of degradation would lead to the accumulation of dysfunctional proteins and organelles, as well as to axonal dystrophy. As such, it is not surprising that these features are more prominent in age-related diseases like AD, where autophagic vesicles accumulate within dystrophic neurites near senile plaques [66,67,68]. Accordingly, upregulation of autophagic degradation in some animal models of different neurodegenerative diseases has been seen to have protective effects [50,65,69,70]. Here, we have focused on the strategies employed to modulate autophagy in mouse models of AD, one of the most studied autophagy-related proteinopathies (again, we refer the reader to a recent review to obtain information on the role of autophagy in other neurodegenerative diseases: [65]).

## 5. Alzheimer’s Disease (AD)

Alzheimer’s disease is the most common neurodegenerative disorder and is considered a proteinopathy that is characterised by the accumulation of deposits of aggregated amyloid-beta (Aβ: *Senile plaques*) and hyperphosphorylated tau (*Tangles*). There are data indicating that autophagy is impaired in this pathology and the accumulation of autophagic vesicles within dystrophic neurites in the brain suggests a progressive deregulation of protein turnover. Several factors associate AD with dysfunctions in autophagy, such as the accumulation of amyloid and tau aggregates that might saturate the clearance system, or mutations in PS1 and PICALM that could affect lysosome activity or different steps in autophagy, respectively [60,65,71]. Alternatively, mTOR hyperactivation in AD models seems to be caused by Aβ hindering autophagy and consequently, contributing to the accumulation of Aβ and tau [72,73].

Autophagic markers like LC3-II, p62 or NBR1 have been reported to be modified in postmortem brains from AD patients and in AD animal models. Numerous studies in mice models of AD have shown a relationship between autophagy and the hallmarks of AD, Aβ or tau aggregates. Genetic deletion of the pro-autophagic protein Becn-1 aggravates the AD pathology, whereas its enhancement or an improvement in lysosomal function reduces the amyloid load (Table 1: [74,75,76,77]. Conversely, pharmacological activation of autophagy has been reported to have positive effects in several AD mouse models (Table 1). Rapamycin is the most often used inducer of autophagy and inhibits mTORC1 in vivo. In our hands, rapamycin reduces amyloid levels (unpublished data), as observed in other mouse models of AD [72,78,79], although contrasting results not related to autophagy have also been described [80]. In recent years, many compounds that induce autophagy or improve lysosomal clearance have shown promising therapeutic benefits in diverse AD mouse models, and some have already been FDA approved (reviewed in Table 1, though surely incomplete). Some of these also induce AMPK/mTORC1-dependent autophagy, such as latrepirdine [81] or resveratrol [82], whereas others promote mTORC1-independent autophagy, for example through the inhibition of HDAC6 with Tubastatin A/ACY-1215 [83], the inhibition of Calpain with Calpeptin or BDA-410 [84], or the inhibition of some tyrosine kinases [85,86].

In addition to the induction of autophagy, the restoration of lysosomal activity is another related therapeutic target for age-associated proteinopathies. Accordingly, some compounds have improved the clearance of amyloid deposits by enhancing the activity of lysosomal proteases, for example Cathepsin B [87], or by restoring acidification through GSK3 inhibition [88]. While most of the data available suggests that enhancing autophagy may improve amyloid degradation, an associated rise in amyloid production has also been described (Table 1). According to this hypothesis, an increase in autophagic vesicle load confers an optimal environment for amyloidogenic processing of amyloid precursor protein (APP) [2,89]. Accordingly, we think that the data currently available suggest that more work should be carried out to clarify whether alternative methods to enhance neuronal autophagy may have additional therapeutic benefits in pathologies like AD or similar proteinopathies.

Cortical neurons from mice were treated with rapamycin 3 h after plating, and they were maintained in the presence of rapamycin for short (30 min and 60 min: “A”) or long periods (32 h: “B”), using DMSO alone as a control. Cell extracts were obtained and analysed in Western blots probed with antibodies against some elements of the Akt-mTORC1 pathway, phosphorylated and total proteins such as Akt, S6K1, S6, etc. GAPDH and beta-actin were used as controls of loading. Note that Akt-pSer472/3 is still evident 30 min or 60 min after rapamycin inhibition (asterisk in A), whereas it is almost completely lost after 32 h in the presence of rapamycin (arrows in B).

## 6. Conclusions

We try to describe and discuss some methods available to monitor autophagy in neurons and in the mammalian brain taking into account the troubles to correlate brain to primary neurons. We dedicated this review primarily on mammalian macroauophagy that we consider highly important in many neuropathologies, such as Parkinson, Alzheimer’s disease or Huntington where the accumulation of peptide/protein strongly suggest an autophagy dysfunction. We focused our review around the hypothesis that enhanced autophagy may be able to provide therapeutic benefits in Alzheimer’s disease, considering this pathology as one of the most prevalent proteinopathies. 

The data summarized strongly suggested that we are far away for understanding of how autophagy is regulated in neurons or in the brain. We have clear data that in some cases, such as rapamycin, the therapeutic effect on amyloidosis reported may be via of mTROC1 inhibition/autophagy enhanced however not all compound modifying mTORC1 activity appear to directly correlated with similar effects. Finally more work have to be done to clarify whether the modification of autophagy always correlated with a reduction on amyloidosis.

## Figures and Tables

**Table 1:** Summary of data using different compounds in AD models. The putative mechanism, connecting or not autophagy, and the therapeutic effects were indicated.

AD Mouse Model	Treatment	Molecular Mechanism	Phenotypic Effect	Ref.
T41 mice (mThy-1-hAPP751; APP V171I,K670M/N671L)	Genetic deletion of Becn-1	Reduced autophagy	Increased amyloid-β levels and deposits. Increased neurodegeneration.	[74]
	Lentivirus-mediated expression of Becn-1		Reduced Aβ levels	
hAPP-J20 mice	Genetic deletion of cystatin C	Increased lysosomal cysteine proteases activity Rescued autophagic-lysosomal pathology	Reduced Aβ levels and Aβ-associated cognitive deficits and behavioural abnormalities	[75]
TgCRND8 mice (APP K670M, M671L, V717F)	Genetic deletion of cystatin B	Increased lysosomal cysteine proteases activity Rescued autophagic-lysosomal pathology	Reduced amyloid-β40 and -β42 levels and prevented the development of learning and memory deficits	[76]
hAPP-J20 mice	Lentivirus-mediated expression of Cathepsin B	C-terminal truncation of Aβ42	Reduced pre-existing amyloid deposits	[77]
APP/PS1 (B6C3-Tg(APPswe, PSEN1dE9)85Dbo/J)	Genetic deletion of one allele of Cathepsin D	Non reduced APP processing or degradation	No changes in Aβ levels	[90]
3× Tg-AD mice (APPSwe /tauP301L/ PS1 knock-in)	Rapamycin	mTORC1 inhibition autophagy induction	Improved learning and memory deficits and reduced amyloid-β and tau pathology	[72]
PDAPP mice (hAPP(J20))	Rapamycin	mTORC1 inhibition autophagy induction chaperone levels increase	Improved cognitive deficits and decreased amyloid-β42 levels	[78,79]
Tg2576 mice	Rapamycin	mTORC1 inhibition inhibition of ADAM10	Increased amyloid-β levels	[80]
TgCRND8 mice (APP K670M, M671L, V717F)	Latrepirdine	mTORC1 inhibition autophagy induction	Improved learning behaviour and reduced amyloid-β42 and a-synuclein levels	[81]
APP/PS1 (B6C3-Tg(APPswe, PSEN1dE9)85Dbo/J)	Resveratrol	AMPK activation Autophagy induction	Reduced amyloid-β40 and -β42 levels	[82]
APPswe/PS1dE9	Tubastatin A and ACY-1215	HDAC6 inhibition facilitated autophagic clearance of Aβ and hyperphosphorylated tau	alleviated behavioural deficits, altered Aβ load, and reduced tau hyperphosphorylation	[83]
APP/PS1 (B6C3-Tg(APPswe, PSEN1dE9)85Dbo/J) APPSwInd	Z-Phe-Ala-diazomethylketone (PADK)	Increased Cathepsin B protein levels and activity Truncation of Aβ42	Reduced Aβ levels and Aβ-associated behavioural and synaptic deficits	[87]
APP/PS1 mice (APP K670M and M671L; PS1 M146L)	BDA-410	Calpain inhibition * Restoration of normal phosphorylation levels of CREB	Improved spatial-working and associative fear memories	[84]
Tg6799 (APP K670N/M671L, I716V, V717I; PS1 M146L/L286V)	L803-mts	GSK3 a/b inhibition Restoration of lysosomal acidification	Reduced Aβ burdens	[88]
AβPPSwe/PS1A246E	Lithium	GSK3β inhibition Attenuated the autophagy activation, reducing APP processing	Reduced Aβ total levels and deposits. Improved spatial learning and memory abilities.	[91]
APP/PS1 mice	Trehalose	Altered the conformation Aβ to prevent its interaction with membranes Autophagy induction	Reduced Aβ deposits, and total Aβ40 levels. Improved spatial memory and learning ability.	[92]
P301S tau mice	Trehalose	Autophagy induction	Reduced tau levels and deposits.	[93]
Tg2576 mice (APP Swe, K670N, M671L)	Trehalose	Autophagy induction	Reduced Aβ and tau deposits.	[94]
Tg6799 mice (APP K670N/M671L, I716V, V717I; PS1 M146L/L286V)	Metformin	AMPK activation and mTORC1 inhibition induces an abnormal accumulation of autophagosomes, promoting APP processing	Increased generation of Aβ	[84]
Tg-APP mice (APP 770 isoform K670N/M671L, E693Q, D694N)	Tyrosine kinase inhibitors (TKIs) (nilotinib, bosutinib)	Increased parkin-Beclin1 interaction Autophagy induction	Reduced Aβ levels. Cognitive improvement.	[85,86]
3 × Tg-AD mice (APPSwe/tau P301L/PS1 M146V)	Nicotinamide	Increased NAD+ biosynthesis, promoting autophagy and lysosomal acidification	Reduced Aβ and hyperphospholylated tau levels. Cognitive improvement.	[95]
APP/PS1 mice (APP K594N/M595L; PS1-dE9)	3-benzyl-5-((2-nitrophenoxy) methyl)-dihydrofuran- 2(3 H)-one (3BDO)	Increased levels of insulin degrading enzyme and neprilysin. Suppressed autophagy via mTOR pathway	Reduced Aβ levels. Prevented AD-like cognitive deficits.	[96]
APP/PS1 mice (APP Swe/PSEN1dE9)	Carbamazepine	Autophagy induction unlikely via mTOR inhibition	Reduced amyloid plaque burden and Aβ42 levels. Alleviated spatial learning and memory deficits.	[97]
3× Tg-AD mice (APPSwe/tau P301L/PS1 M146V)	GTM-1	Autophagy induction	Reduced Aβ levels and deposition. Alleviated spatial learning and memory deficits.	[98]
APP-OSK mice Tg2576 mice Tau609 mice	Rifampicin	Inhibition of oligomer formation preventing protein accumulation and autophagy dysfunction.	Reduced Aβ oligomer accumulation and tau hyperphosphorylation. Improved spatial memory.	[99]
APP/PS1 mice (APP Swe/PSEN1dE9)	LX2343	Suppression of JNK-mediated phosphorylation of APP(Thr668), and thus APP processing. PI3K/AKT/mTOR inhibition and induction of autophagy.	Reduced Aβ levels and deposits. Alleviated spatial learning and memory deficits.	[100]
3 × Tg-AD mice (APPSwe/tau P301L/PS1 M146V)	Berberine	Autophagy induction.	Reduced Aβ levels and deposits. Improved spatial learning capacity and memory retention.	[101]
3× Tg-AD mice (APPSwe/tau P301L/PS1 M146V)	Selenomethionine (Se-Met)	Akt activation and GSK3β inhibition. Autophagy induction (AMPK-mTORC1 dependent).	Reduced total and hyper-phosphorylated tau. Improved spatial memory	[102]

* Calpain inhibition has been described as an mTORC1-independent mechanism to induce autophagy [103,104,105], although no relationship has been studied in this report.

## References

[1] Rubinsztein D.C. (2006). The roles of intracellular protein-degradation pathways in neurodegeneration. Nature.

[2] Yu W.H., Cuervo A.M., Kumar A., Peterhoff C.M., Schmidt S.D., Lee J.H., Mohan P.S., Mercken M., Farmery M.R., Tjernberg L.O. (2005). Macroautophagy-a novel Beta-amyloid peptide-generating pathway activated in Alzheimer’s disease. J. Cell Biol..

[3] Nixon R.A., Wegiel J., Kumar A., Yu W.H., Peterhoff C., Cataldo A., Cuervo A.M. (2005). Extensive involvement of autophagy in Alzheimer disease: An immuno-electron microscopy study. J. Neuropathol. Exp. Neurol..

[4] Levine B., Kroemer G. (2008). Autophagy in the pathogenesis of disease. Cell.

[5] Mizushima N., Levine B., Cuervo A.M., Klionsky D.J. (2008). Autophagy fights disease through cellular self-digestion. Nature.

[6] Suzuki K., Terry R.D. (1967). Fine structural localization of acid phosphatase in senile plaques in Alzheimer’s presenile dementia. Acta Neuropathol..

[7] Boya P., Reggiori F., Codogno P. (2013). Emerging regulation and functions of autophagy. Nat. Cell Biol..

[8] Kabeya Y., Mizushima N., Ueno T., Yamamoto A., Kirisako T., Noda T., Kominami E., Ohsumi Y., Yoshimori T. (2000). LC3, a mammalian homologue of yeast Apg8p, is localized in autophagosome membranes after processing. EMBO J..

[9] Kabeya Y., Mizushima N., Yamamoto A., Oshitani-Okamoto S., Ohsumi Y., Yoshimori T. (2004). LC3, GABARAP and GATE16 localize to autophagosomal membrane depending on form-II formation. J. Cell Sci..

[10] Rubinsztein D.C., Shpilka T., Elazar Z. (2012). Mechanisms of autophagosome biogenesis. Curr. Biol..

[11] Johansen T., Lamark T. (2011). Selective autophagy mediated by autophagic adapter proteins. Autophagy.

[12] Kirkin V., Lamark T., Sou Y.S., Bjorkoy G., Nunn J.L., Bruun J.A., Shvets E., McEwan D.G., Clausen T.H., Wild P. (2009). A role for NBR1 in autophagosomal degradation of ubiquitinated substrates. Mol. Cell.

[13] Klionsky D.J., Abdelmohsen K., Abe A., Abedin M.J., Abeliovich H., Acevedo-Arozena A., Adachi H., Adams C.M., Adams P.D., Adeli K. (2016). Guidelines for the use and interpretation of assays for monitoring autophagy (3rd edition). Autophagy.

[14] Komatsu M., Kominami E., Tanaka K. (2006). Autophagy and neurodegeneration. Autophagy.

[15] Lee S., Sato Y., Nixon R.A. (2011). Lysosomal proteolysis inhibition selectively disrupts axonal transport of degradative organelles and causes an Alzheimer’s-like axonal dystrophy. J. Neurosci..

[16] Maiuri M.C., Zalckvar E., Kimchi A., Kroemer G. (2007). Self-eating and self-killing: Crosstalk between autophagy and apoptosis. Nat. Rev. Mol. Cell Biol..

[17] Hara T., Nakamura K., Matsui M., Yamamoto A., Nakahara Y., Suzuki-Migishima R., Yokoyama M., Mishima K., Saito I., Okano H. (2006). Suppression of basal autophagy in neural cells causes neurodegenerative disease in mice. Nature.

[18] Herculano-Houzel S. (2014). The glia/neuron ratio: How it varies uniformly across brain structures and species and what that means for brain physiology and evolution. Glia.

[19] Bentley M., Banker G. (2016). The cellular mechanisms that maintain neuronal polarity. Nat. Rev. Neurosci..

[20] Schelski M., Bradke F. (2017). Neuronal polarization: From spatiotemporal signaling to cytoskeletal dynamics. Mol. Cell Neurosci..

[21] Yang D.S., Stavrides P., Saito M., Kumar A., Rodriguez-Navarro J.A., Pawlik M., Huo C., Walkley S.U., Saito M., Cuervo A.M. (2014). Defective macroautophagic turnover of brain lipids in the TgCRND8 Alzheimer mouse model: Prevention by correcting lysosomal proteolytic deficits. Brain.

[22] Wang D.B., Kinoshita Y., Kinoshita C., Uo T., Sopher B.L., Cudaback E., Keene C.D., Bilousova T., Gylys K., Case A. (2015). Loss of endophilin-B1 exacerbates Alzheimer’s disease pathology. Brain.

[23] Ariosa A.R., Klionsky D.J. (2016). Autophagy core machinery: Overcoming spatial barriers in neurons. J. Mol. Med. (Berlin).

[24] Maday S., Holzbaur E.L. (2014). Autophagosome biogenesis in primary neurons follows an ordered and spatially regulated pathway. Dev. Cell.

[25] Maday S., Holzbaur E.L. (2016). Compartment-Specific Regulation of Autophagy in Primary Neurons. J. Neurosci..

[26] Maday S., Holzbaur E.L. (2012). Autophagosome assembly and cargo capture in the distal axon. Autophagy.

[27] Ashrafi G., Schlehe J.S., LaVoie M.J., Schwarz T.L. (2014). Mitophagy of damaged mitochondria occurs locally in distal neuronal axons and requires PINK1 and Parkin. J. Cell Biol..

[28] Yusuf M., Leung K., Morris K.J., Volpi E.V. (2013). Comprehensive cytogenomic profile of the in vitro neuronal model SH-SY5Y. Neurogenetics.

[29] Tsvetkov A.S., Mitra S., Finkbeiner S. (2009). Protein turnover differences between neurons and other cells. Autophagy.

[30] Klionsky D.J., Abdalla F.C., Abeliovich H., Abraham R.T., Acevedo-Arozena A., Adeli K., Agholme L., Agnello M., Agostinis P., Aguirre-Ghiso J.A. (2012). Guidelines for the use and interpretation of assays for monitoring autophagy. Autophagy.

[31] Sarbassov D.D., Ali S.M., Sengupta S., Sheen J.H., Hsu P.P., Bagley A.F., Markhard A.L., Sabatini D.M. (2006). Prolonged rapamycin treatment inhibits mTORC2 assembly and Akt/PKB. Mol. Cell.

[32] Diez H., Garrido J.J., Wandosell F. (2012). Specific roles of Akt iso forms in apoptosis and axon growth regulation in neurons. PLoS ONE.

[33] Diez H., Benitez M.J., Fernandez S., Torres-Aleman I., Garrido J.J., Wandosell F. (2016). Class I PI3-kinase or Akt inhibition do not impair axonal polarization, but slow down axonal elongation. Biochim. Biophys. Acta.

[34] Li M., Khambu B., Zhang H., Kang J.H., Chen X., Chen D., Vollmer L., Liu P.Q., Vogt A., Yin X.M. (2013). Suppression of lysosome function induces autophagy via a feedback down-regulation of MTOR complex 1 (MTORC1) activity. J. Biol. Chem..

[35] Deitch J.S., Banker G.A. (1993). An electron microscopic analysis of hippocampal neurons developing in culture: Early stages in the emergence of polarity. J. Neurosci..

[36] Blomquist E., Fredriksson B.A., Brunk U. (1980). Electron probe X-ray microanalysis of residual bodies in aged cultured human glial cells. Ultrastruct. Pathol..

[37] Ni H.M., Bockus A., Wozniak A.L., Jones K., Weinman S., Yin X.M., Ding W.X. (2011). Dissecting the dynamic turnover of GFP-LC3 in the autolysosome. Autophagy.

[38] Kimura S., Noda T., Yoshimori T. (2007). Dissection of the autophagosome maturation process by a novel reporter protein, tandem fluorescent-tagged LC3. Autophagy.

[39] Novak I., Kirkin V., McEwan D.G., Zhang J., Wild P., Rozenknop A., Rogov V., Lohr F., Popovic D., Occhipinti A. (2010). Nix is a selective autophagy receptor for mitochondrial clearance. EMBO Rep..

[40] Schwarten M., Mohrluder J., Ma P., Stoldt M., Thielmann Y., Stangler T., Hersch N., Hoffmann B., Merkel R., Willbold D. (2009). Nix directly binds to GABARAP: A possible crosstalk between apoptosis and autophagy. Autophagy.

[41] Koike M., Tanida I., Nanao T., Tada N., Iwata J., Ueno T., Kominami E., Uchiyama Y. (2013). Enrichment of GABARAP relative to LC3 in the axonal initial segments of neurons. PLoS ONE.

[42] Davis C.H., Kim K.Y., Bushong E.A., Mills E.A., Boassa D., Shih T., Kinebuchi M., Phan S., Zhou Y., Bihlmeyer N.A. (2014). Transcellular degradation of axonal mitochondria. Proc. Natl. Acad. Sci. USA.

[43] Mizushima N., Yamamoto A., Matsui M., Yoshimori T., Ohsumi Y. (2004). In vivo analysis of autophagy in response to nutrient starvation using transgenic mice expressing a fluorescent autophagosome marker. Mole. Biol. Cell.

[44] Chu C.T., Plowey E.D., Dagda R.K., Hickey R.W., Cherra S.J., Clark R.S. (2009). Autophagy in neurite injury and neurodegeneration: In vitro and in vivo models. Method. Enzymol..

[45] Esteban-Martinez L., Boya P. (2015). Autophagic flux determination in vivo and ex vivo. Methods.

[46] Zhan L., Liu L., Li K., Wu B., Liu D., Liang D., Wen H., Wang Y., Sun W., Liao W. (2016). Neuroprotection of hypoxic postconditioning against global cerebral ischemia through influencing posttranslational regulations of heat shock protein 27 in adult rats. Brain Pathol..

[47] Perry C.N., Kyoi S., Hariharan N., Takagi H., Sadoshima J., Gottlieb R.A. (2009). Novel methods for measuring cardiac autophagy in vivo. Method. Enzymol..

[48] Carloni S., Girelli S., Scopa C., Buonocore G., Longini M., Balduini W. (2010). Activation of autophagy and Akt/CREB signaling play an equivalent role in the neuroprotective effect of rapamycin in neonatal hypoxia-ischemia. Autophagy.

[49] Sarkar C., Zhao Z., Aungst S., Sabirzhanov B., Faden A.I., Lipinski M.M. (2014). Impaired autophagy flux is associated with neuronal cell death after traumatic brain injury. Autophagy.

[50] Ahmed I., Liang Y., Schools S., Dawson V.L., Dawson T.M., Savitt J.M. (2012). Development and characterization of a new Parkinson’s disease model resulting from impaired autophagy. J. Neurosci..

[51] Nishiyama J., Miura E., Mizushima N., Watanabe M., Yuzaki M. (2007). Aberrant membranes and double-membrane structures accumulate in the axons of Atg5-null Purkinje cells before neuronal death. Autophagy.

[52] Orvedahl A., Levine B. (2008). Viral evasion of autophagy. Autophagy.

[53] Komatsu M., Waguri S., Chiba T., Murata S., Iwata J., Tanida I., Ueno T., Koike M., Uchiyama Y., Kominami E. (2006). Loss of autophagy in the central nervous system causes neurodegeneration in mice. Nature.

[54] Liang C.C., Wang C., Peng X., Gan B., Guan J.L. (2010). Neural-specific deletion of FIP200 leads to cerebellar degeneration caused by increased neuronal death and axon degeneration. J. Bio. Chem..

[55] Orosco L.A., Ross A.P., Cates S.L., Scott S.E., Wu D., Sohn J., Pleasure D., Pleasure S.J., Adamopoulos I.E., Zarbalis K.S. (2014). Loss of Wdfy3 in mice alters cerebral cortical neurogenesis reflecting aspects of the autism pathology. Nat. Commun..

[56] Joo J.H., Wang B., Frankel E., Ge L., Xu L., Iyengar R., Li-Harms X., Wright C., Shaw T.I., Lindsten T. (2016). The Noncanonical Role of ULK/ATG1 in ER-to-Golgi Trafficking is Essential for Cellular Homeostasis. Mol. Cell.

[57] Zhao Y.G., Sun L., Miao G., Ji C., Zhao H., Sun H., Miao L., Yoshii S.R., Mizushima N., Wang X. (2015). The autophagy gene Wdr45/Wipi4 regulates learning and memory function and axonal homeostasis. Autophagy.

[58] Castillo K., Valenzuela V., Matus S., Nassif M., Onate M., Fuentealba Y., Encina G., Irrazabal T., Parsons G., Court F.A. (2013). Measurement of autophagy flux in the nervous system in vivo. Cell Death Dis..

[59] Matus S., Valenzuela V., Hetz C. (2014). A new method to measure autophagy flux in the nervous system. Autophagy.

[60] Lee J.H., Yu W.H., Kumar A., Lee S., Mohan P.S., Peterhoff C.M., Wolfe D.M., Martinez-Vicente M., Massey A.C., Sovak G. (2010). Lysosomal proteolysis and autophagy require presenilin 1 and are disrupted by Alzheimer-related PS1 mutations. Cell.

[61] Ebrahimi-Fakhari D., Wahlster L., Hoffmann G.F., Kolker S. (2014). Emerging role of autophagy in pediatric neurodegenerative and neurometabolic diseases. Pediatr. Res..

[62] Ginet V., Spiehlmann A., Rummel C., Rudinskiy N., Grishchuk Y., Luthi-Carter R., Clarke P.G., Truttmann A.C., Puyal J. (2014). Involvement of autophagy in hypoxic-excitotoxic neuronal death. Autophagy.

[63] Puyal J., Ginet V., Grishchuk Y., Truttmann A.C., Clarke P.G. (2012). Neuronal autophagy as a mediator of life and death: Contrasting roles in chronic neurodegenerative and acute neural disorders. Neuroscientist.

[64] Ginet V., Pittet M.P., Rummel C., Osterheld M.C., Meuli R., Clarke P.G., Puyal J., Truttmann A.C. (2014). Dying neurons in thalamus of asphyxiated term newborns and rats are autophagic. Ann. Neurol..

[65] Menzies F.M., Fleming A., Caricasole A., Bento C.F., Andrews S.P., Ashkenazi A., Fullgrabe J., Jackson A., Jimenez-Sanchez M., Karabiyik C. (2017). Autophagy and Neurodegeneration: Pathogenic Mechanisms and Therapeutic Opportunities. Neuron.

[66] Lipinski M.M., Zheng B., Lu T., Yan Z., Py B.F., Ng A., Xavier R.J., Li C., Yankner B.A., Scherzer C.R. (2010). Genome-wide analysis reveals mechanisms modulating autophagy in normal brain aging and in Alzheimer’s disease. Proc. Natl. Acad. Sci. USA.

[67] Nixon R.A. (2007). Autophagy, amyloidogenesis and Alzheimer disease. J. Cell Sci..

[68] Boland B., Kumar A., Lee S., Platt F.M., Wegiel J., Yu W.H., Nixon R.A. (2008). Autophagy induction and autophagosome clearance in neurons: Relationship to autophagic pathology in Alzheimer’s disease. J. Neurosci..

[69] Dupuis L. (2014). Mitochondrial quality control in neurodegenerative diseases. Biochimie.

[70] Ghavami S., Shojaei S., Yeganeh B., Ande S.R., Jangamreddy J.R., Mehrpour M., Christoffersson J., Chaabane W., Moghadam A.R., Kashani H.H. (2014). Autophagy and apoptosis dysfunction in neurodegenerative disorders. Prog. Neurobiol..

[71] Bagyinszky E., Youn Y.C., An S.S., Kim S. (2014). The genetics of Alzheimer’s disease. Clin. Interv. Aging.

[72] Caccamo A., Majumder S., Richardson A., Strong R., Oddo S. (2010). Molecular interplay between mammalian target of rapamycin (mTOR), amyloid-beta, and Tau: Effects on cognitive impairments. J. Biol. Chem..

[73] Caccamo A., Maldonado M.A., Majumder S., Medina D.X., Holbein W., Magri A., Oddo S. (2011). Naturally secreted amyloid-beta increases mammalian target of rapamycin (mTOR) activity via a PRAS40-mediated mechanism. J. Bio. Chem..

[74] Pickford F., Masliah E., Britschgi M., Lucin K., Narasimhan R., Jaeger P.A., Small S., Spencer B., Rockenstein E., Levine B. (2008). The autophagy-related protein beclin 1 shows reduced expression in early Alzheimer disease and regulates amyloid beta accumulation in mice. J. Clin. Investig..

[75] Sun B., Zhou Y., Halabisky B., Lo I., Cho S.H., Mueller-Steiner S., Devidze N., Wang X., Grubb A., Gan L. (2008). Cystatin C-cathepsin B axis regulates amyloid beta levels and associated neuronal deficits in an animal model of Alzheimer’s disease. Neuron.

[76] Yang D.S., Stavrides P., Mohan P.S., Kaushik S., Kumar A., Ohno M., Schmidt S.D., Wesson D., Bandyopadhyay U., Jiang Y. (2011). Reversal of autophagy dysfunction in the TgCRND8 mouse model of Alzheimer’s disease ameliorates amyloid pathologies and memory deficits. Brain.

[77] Mueller-Steiner S., Zhou Y., Arai H., Roberson E.D., Sun B., Chen J., Wang X., Yu G., Esposito L., Mucke L. (2006). Antiamyloidogenic and neuroprotective functions of cathepsin B: Implications for Alzheimer’s disease. Neuron.

[78] Spilman P., Podlutskaya N., Hart M.J., Debnath J., Gorostiza O., Bredesen D., Richardson A., Strong R., Galvan V. (2010). Inhibition of mTOR by rapamycin abolishes cognitive deficits and reduces amyloid-beta levels in a mouse model of Alzheimer’s disease. PLoS ONE.

[79] Pierce A., Podlutskaya N., Halloran J.J., Hussong S.A., Lin P.Y., Burbank R., Hart M.J., Galvan V. (2013). Over-expression of heat shock factor 1 phenocopies the effect of chronic inhibition of TOR by rapamycin and is sufficient to ameliorate Alzheimer’s-like deficits in mice modeling the disease. J. Neurochem..

[80] Zhang S., Salemi J., Hou H., Zhu Y., Mori T., Giunta B., Obregon D., Tan J. (2010). Rapamycin promotes beta-amyloid production via ADAM-10 inhibition. Biochem. Biophys. Res. Commun..

[81] Steele J.W., Lachenmayer M.L., Ju S., Stock A., Liken J., Kim S.H., Delgado L.M., Alfaro I.E., Bernales S., Verdile G. (2013). Latrepirdine improves cognition and arrests progression of neuropathology in an Alzheimer’s mouse model. Mol. Psychiatry.

[82] Vingtdeux V., Giliberto L., Zhao H., Chandakkar P., Wu Q., Simon J.E., Janle E.M., Lobo J., Ferruzzi M.G., Davies P. (2010). AMP-activated protein kinase signaling activation by resveratrol modulates amyloid-beta peptide metabolism. J. Biol. Chem..

[83] Zhang L., Liu C., Wu J., Tao J.J., Sui X.L., Yao Z.G., Xu Y.F., Huang L., Zhu H., Sheng S.L. (2014). Tubastatin A/ACY-1215 improves cognition in Alzheimer’s disease transgenic mice. J. Alzheimer’s Dis..

[84] Trinchese F., Fa M., Liu S., Zhang H., Hidalgo A., Schmidt S.D., Yamaguchi H., Yoshii N., Mathews P.M., Nixon R.A. (2008). Inhibition of calpains improves memory and synaptic transmission in a mouse model of Alzheimer disease. J. Clin. Investig..

[85] Lonskaya I., Hebron M.L., Desforges N.M., Franjie A., Moussa C.E. (2013). Tyrosine kinase inhibition increases functional parkin-Beclin-1 interaction and enhances amyloid clearance and cognitive performance. EMBO Mol. Med..

[86] Lonskaya I., Hebron M.L., Desforges N.M., Schachter J.B., Moussa C.E. (2014). Nilotinib-induced autophagic changes increase endogenous parkin level and ubiquitination, leading to amyloid clearance. J. Mol. Med. (Berlin).

[87] Butler D., Hwang J., Estick C., Nishiyama A., Kumar S.S., Baveghems C., Young-Oxendine H.B., Wisniewski M.L., Charalambides A., Bahr B.A. (2011). Protective effects of positive lysosomal modulation in Alzheimer’s disease transgenic mouse models. PLoS ONE.

[88] Avrahami L., Farfara D., Shaham-Kol M., Vassar R., Frenkel D., Eldar-Finkelman H. (2013). Inhibition of glycogen synthase kinase-3 ameliorates beta-amyloid pathology and restores lysosomal acidification and mammalian target of rapamycin activity in the Alzheimer disease mouse model: In vivo and in vitro studies. J. Biol. Chem..

[89] Son S.M., Shin H.J., Byun J., Kook S.Y., Moon M., Chang Y.J., Mook-Jung I. (2016). Metformin Facilitates Amyloid-beta Generation by beta- and gamma-Secretases via Autophagy Activation. J. Alzheimer’s Dis..

[90] Cheng S., Wani W.Y., Hottman D.A., Jeong A., Cao D., LeBlanc K.J., Saftig P., Zhang J. (2017). Haplodeficiency of Cathepsin D does not affect cerebral amyloidosis and autophagy in APP/PS1 transgenic mice. J. Nerochem..

[91] Zhang X., Heng X., Li T., Li L., Yang D., Zhang X., Du Y., Doody R.S., Le W. (2011). Long-term treatment with lithium alleviates memory deficits and reduces amyloid-beta production in an aged Alzheimer’s disease transgenic mouse model. J. Alzheimer’s Dis..

[92] Du J., Liang Y., Xu F., Sun B., Wang Z. (2013). Trehalose rescues Alzheimer’s disease phenotypes in APP/PS1 transgenic mice. J. Pharm. Pharmacol..

[93] Schaeffer V., Lavenir I., Ozcelik S., Tolnay M., Winkler D.T., Goedert M. (2012). Stimulation of autophagy reduces neurodegeneration in a mouse model of human tauopathy. Brain.

[94] Perucho J., Casarejos M.J., Gomez A., Solano R.M., de Yebenes J.G., Mena M.A. (2012). Trehalose protects from aggravation of amyloid pathology induced by isoflurane anesthesia in APP(swe) mutant mice. Curr. Alzheimer Res..

[95] Liu D., Pitta M., Jiang H., Lee J.H., Zhang G., Chen X., Kawamoto E.M., Mattson M.P. (2013). Nicotinamide forestalls pathology and cognitive decline in Alzheimer mice: Evidence for improved neuronal bioenergetics and autophagy procession. Neurobiol. Aging.

[96] Wei L., Yang H., Xie Z., Yang S., Yang H., Zhao C., Wang P., Xu S., Miao J., Zhao B. (2012). A butyrolactone derivative 3BDO alleviates memory deficits and reduces amyloid-beta deposition in an AbetaPP/PS1 transgenic mouse model. J. Alzheimer’s Dis..

[97] Li L., Zhang S., Zhang X., Li T., Tang Y., Liu H., Yang W., Le W. (2013). Autophagy enhancer carbamazepine alleviates memory deficits and cerebral amyloid-beta pathology in a mouse model of Alzheimer’s disease. Curr. Alzheimer Res..

[98] Chu C., Zhang X., Ma W., Li L., Wang W., Shang L., Fu P. (2013). Induction of autophagy by a novel small molecule improves abeta pathology and ameliorates cognitive deficits. PLoS ONE.

[99] Umeda T., Ono K., Sakai A., Yamashita M., Mizuguchi M., Klein W.L., Yamada M., Mori H., Tomiyama T. (2016). Rifampicin is a candidate preventive medicine against amyloid-beta and tau oligomers. Brain.

[100] Guo X.D., Sun G.L., Zhou T.T., Xu X., Zhu Z.Y., Rukachaisirikul V., Hu L.H., Shen X. (2016). Small molecule LX2343 ameliorates cognitive deficits in AD model mice by targeting both amyloid beta production and clearance. Acta Pharmacol. Sin..

[101] Huang M., Jiang X., Liang Y., Liu Q., Chen S., Guo Y. (2017). Berberine improves cognitive impairment by promoting autophagic clearance and inhibiting production of beta-amyloid in APP/tau/PS1 mouse model of Alzheimer’s disease. Exp. Gerontol..

[102] Zhang Z.H., Wu Q.Y., Zheng R., Chen C., Chen Y., Liu Q. (2017). Selenomethionine Mitigates Cognitive Decline by Targeting Both Tau Hyperphosphorylation and Autophagic Clearance in an Alzheimer’s Disease Mouse Model. J. Neurosci..

[103] Sarkar S., Ravikumar B., Floto R.A., Rubinsztein D.C. (2009). Rapamycin and mTOR-independent autophagy inducers ameliorate toxicity of polyglutamine-expanded huntingtin and related proteinopathies. Cell Death Differ..

[104] Xia H.G., Zhang L., Chen G., Zhang T., Liu J., Jin M., Ma X., Ma D., Yuan J. (2010). Control of basal autophagy by calpain1 mediated cleavage of ATG5. Autophagy.

[105] Mestre M.B., Colombo M.I. (2012). cAMP and EPAC are key players in the regulation of the signal transduction pathway involved in the alpha-hemolysin autophagic response. PLoS Pathog..

